# Quality of Conventional versus Artificial Intelligence Oral Surgery Consent Forms: Comparative Analysis

**DOI:** 10.2196/59851

**Published:** 2026-01-05

**Authors:** Jan Gaessler, Bernhard Remschmidt, Ann-Kathrin Jopp, Behrouz Arefnia, Adrian Franke, Marcus Rieder

**Affiliations:** 1 Division of Oral and Maxillofacial Surgery, Department of Dental Medicine and Oral Health, Medical University of Graz, Auenbruggerplatz 5/6, Graz, 8032, Austria, 43 316 385 83500; 2 Clinical psychologist and psychotherapist in private practice, Hannover, Germany; 3 Division of Restorative Dentistry, Periodontology and Prosthodontics, Department of Dental Medicine and Oral Health, Medical University of Graz, Graz, Austria; 4 Department of Oral and Maxillofacial Surgery, University Hospital Carl Gustav Carus, Dresden University of Technology, Dresden, Germany

**Keywords:** oral surgical procedures, informed consent, quality control, artificial intelligence, oral surgery, consent form, AI, dental health, oral surgeon, patient care, paitent autonomy, dentistry

## Abstract

Artificial intelligence–generated informed consent forms for oral surgery demonstrated higher quality and better readability than conventional web-based forms, though both fell short of recommended comprehension levels.

## Introduction

Informed consent is a foundational element of ethical and legal medical care, ensuring patients understand the nature, risks, and alternatives of proposed treatments [1,2]. In oral surgery, where procedures can be complex and invasive, clear and high-quality informed consent forms (ICFs) are especially critical. However, many ICFs exceed the recommended 6th-grade reading level, limiting patient comprehension [3]. With the recent rise of artificial intelligence (AI), particularly large language models (LLMs), there is growing interest in their potential to improve patient communication [4,5]. This study aimed to assess the quality and readability of conventional, web-based oral surgery ICFs and compare them to those generated by AI-based LLMs.

## Methods

Ten common oral surgery procedures were selected (ie, apicoectomy, biopsy, bone augmentation, cystectomy, dental implants, incision and drainage, local anesthesia, periodontal surgery, tooth extraction, and wisdom tooth removal). Using Google Chrome in incognito mode, 300 web-based ICFs (ie, 30 per procedure) were collected (see search strategy in Multimedia Appendix 1). In parallel, four LLMs (ChatGPT 3.5, Claude, Bard, and Bing Chat) were prompted to generate ICFs for the same procedures using standardized requests. Per every procedure and LLM, two basic and non-directive prompts were developed to minimize bias and ensure neutrality, resulting in 80 AI-generated ICFs (see Multimedia Appendix 1). Subsequently, two oral and maxillofacial surgeons screened the collected forms using predefined inclusion and exclusion criteria (see Multimedia Appendix 1).

Quality was assessed using a newly developed alteration of the well-established DISCERN instrument [6], namely the Graz Assessment Tool for Written Informed Consent Keypoints (GATWICK; see Multimedia Appendix 1). It was validated through expert review for content relevance and consistency. It includes 11 items scored on a 5-point Likert scale (total score range 11‐55). Two oral and maxillofacial surgery residents independently rated all forms. Readability was evaluated using six established formulas (ie, Automated Readability Index, Coleman-Liau, Flesch-Kincaid, FORCAST, Gunning Fog, and Simple Measure of Gobbledygook), and an average reading grade level was calculated [7]. Statistical analyses included the Mann-Whitney *U* test, Kruskal-Wallis test, and Kendall tau-b, with significance set at *P*≤.05.

## Results

Of 380 screened documents, 213 ICFs met the inclusion criteria: 136 web-based and 77 AI-generated ones. The inter-rater reliability for GATWICK scores was excellent (intraclass correlation coefficient=0.948).

Regarding the quality, AI-generated ICFs had significantly higher total GATWICK scores compared to web-based ones (median 32.5, IQR 28-35.5 vs median 27.5, IQR 20.375-37; *P*=.007). Items related to treatment alternatives, rationale for recommended intervention, and discussion of options scored particularly higher in AI-generated forms. Web-based ICFs scored better in perioperative behavior instructions.

Considering the readability, web-based forms were significantly harder to read (median grade level 12.45, IQR 11.3-13.325) than AI-generated forms (median 10.7, IQR 10.1-12.4; *P*<.001), although neither met the recommended 6th-grade level. Readability was weakly correlated with overall quality (*τ*=0.132; *P*=.005).

The word count was higher for web-based forms (median 794 words, IQR 475.25-1068.75 words) than AI-generated ones (median 338 words, IQR 296-381 words; *P*<.001). Longer forms showed a weak correlation with higher quality (*τ*=0.270; *P*<.001).

Among LLMs, ChatGPT-powered services (ie, ChatGPT 3.5 and Claude) scored significantly higher in terms of quality. ICFs on tooth extraction scored significantly worse when compared with periodontal surgery forms. AI-generated informed consent forms performed significantly better than conventional versions, with notable differences across oral surgical procedures and among the types of LLMs used (Table 1).

**Table 1.** Quality of informed consent forms (ICFs) measured through the GATWICK (Graz Assessment Tool for Written Informed Consent Keypoints) score.

Quality	Median (IQR)	*P* value
Overall quality		.007^a^
Conventional (i.e., web-based) ICFs	27.50 (20.125-37)	
Artificial intelligence-generated ICFs	32.50 (28-36.25)	
All combined	31.00 (23-37)	
Differences by procedure		.004^b^
Apicoectomy	27.00 (21.75-34.875)	
Biopsy	30.50 (25.75-33)	
Oral bone augmentation	31.50 (25.75-37.5)	
Dental cystectomy	31.25 (23-33.875)	
Dental implants	33.25 (20.625-37.125)	
Oral incision and drainage	31.50 (23.5-39.5)	
Dental local anesthesia	28.50 (21-34.5)	
Periodontal surgery	36.50 (32.5-42)	
Tooth extraction	23.50 (20-32.75)	
Wisdom tooth removal	28.25 (20-36.875)	
Differences by large language model		<.001^b^
ChatGPT	34.25 (33-37)	
Claude	40.50 (35-43)	
Bing Chat	30.00 (27.25-31.75)	
Google Bard	26.50 (22.75-31.375)	

a Mann-Whitney *U* test.

b Kruskal-Wallis test.

## Discussion

### Principal Findings

This study found that conventional oral surgery ICFs available online are generally of modest quality and exceed recommended reading levels. AI-generated ICFs outperformed web-based ones in both quality and readability, although they too fell short of ideal readability standards.

These findings are consistent with prior research across medical disciplines, which show that most ICFs are written at a level too advanced for the average patient [8,9]. Notably, AI-generated forms more consistently addressed key informed consent components such as treatment alternatives and rationale, suggesting that LLMs may serve as valuable tools in drafting patient-centered documents. However, AI models may also produce inaccuracies or omit procedure-specific nuances, highlighting the need for expert review [10].

The limitations of this study include its focus on English-language materials and the variability inherent in AI outputs depending on prompt phrasing or model version. While the GATWICK tool demonstrated strong reliability, further validation is needed.

### Conclusion

AI-based LLMs offer a promising avenue for improving the quality and accessibility of oral surgery informed consent documents. Future efforts should focus on refining AI outputs and integrating clinician oversight to ensure accuracy, comprehensiveness, and patient comprehension.

## Supplementary material

10.2196/59851 Multimedia Appendix 1 Methodology showing the utilization of the Boolean operator “OR” helped to broaden the web search as it accounted for differences regarding designation and spelling (i.e., American versus British English). Detailed description of the Graz Assessment Tool of Written Informed Consent Keypoints (GATWICK).

## Abbreviations

AI: Artificial intelligence
GATWICK: Graz Assessment Tool for Written Informed Consent Keypoints
ICF: informed consent form
LLM: large language models
