# Supplementary material for: Quality of Conventional versus Artificial Intelligence Oral Surgery Consent Forms: Comparative Analysis
Source: J Med Internet Res. 2026 Jan 5;28:e59851. doi: 10.2196/59851 (PMC12768391; doi:10.2196/59851)
Supplement: Multimedia Appendix 1 [file jmir-v28-e59851-s001.docx]

**Research Letter**

**Quality of Conventional versus Artificial Intelligence Oral Surgery Consent Forms: a Comparative Analysis**

**Author list:** Jan Gaessler^1,2^; Bernhard Remschmidt^1^; Ann-Kathrin Jopp, Ph.D., M.Sc.^3^; Behrouz Arefnia^4^; Adrian Franke^5^; and Marcus Rieder^1^

1. Division of Oral and Maxillofacial Surgery, Department of Dental Medicine and Oral Health, Medical University of Graz; Auenbruggerplatz 5/6, 8032 Graz, Austria
2. Department of Oral and Maxillofacial Surgery, Hannover Medical School, Carl-Neuberg-Str. 1, 30625 Hannover, Germany
3. Clinical psychologist and psychotherapist in private practice; Schlesierstr. 12, 31139 Hildesheim, Germany
4. Division of Restorative Dentistry, Periodontology and Prosthodontics, Department of Dental Medicine and Oral Health, Medical University of Graz, Billrothgasse 4, 8010 Graz, Austria
5. Department of Oral and Maxillofacial Surgery, University Hospital Carl Gustav Carus, Dresden University of Technology, Fetscherstr. 74, 01307 Dresden, Germany

**Supplemental Files**

| **Methodology** |  | | | |
| --- | --- | --- | --- | --- |
| ***Inclusion/exclusion criteria*** | | | |  |
| - English - Available (i.e., not licensed) | | - Non-English - Scientific publication - Patient information/education material - Video - Wrong topic - Too generic - Duplicate | | |
| ***Search strategy for Web-based informed consent forms*** | | | |  |
| *Apicoectomy* | Apicoectomy OR apicetomy OR root end surgery AND informed consent form | | | |
| *Biopsy* | Oral biopsy OR mouth biopsy AND informed consent form | | | |
| *Oral bone augmentation* | Dental bone graft OR sinus lift AND informed consent form | | | |
| *Dental cystectomy* | Oral cystectomy OR removal of oral and jaw cysts AND informed consent form | | | |
| *Dental implants* | Dental implant surgery OR tooth implant surgery AND informed consent form | | | |
| *Oral incision and drainage* | Dental abscess surgery OR oral incision and drainage AND informed consent form | | | |
| *Dental local anesthesia* | Dental local anesthesia OR dental local anesthesia AND informed consent form | | | |
| *Periodontal surgery* | Periodontal surgery OR gum flap surgery AND informed consent form | | | |
| *Tooth extraction* | Tooth extraction OR tooth removal AND informed consent form | | | |
| *Wisdom tooth removal* | Wisdom teeth surgery OR wisdom teeth removal AND informed consent form | | | |
| ***AI prompts*** |  | | | |
| *“Hello, please provide me with an informed consent form for patients scheduled to undergo [procedure name].”* | | | *Hello, please provide me with an informed consent form for patients scheduled to undergo [procedure name]. It should ideally include information about the diagnosis, the nature and purpose of recommended interventions as well as the burdens, risks, and expected benefits of all options, including forgoing treatment.”* | |

**Supplemental file 1**. Utilization of the Boolean operator “OR” helped to broaden the web search as it accounted for differences regarding designation and spelling (i.e., American versus British English).

| **Graz Assessment Tool of Written Informed Consent Keypoints (GATWICK)** | | | |  |
| --- | --- | --- | --- | --- |
| **Items** |  | | | |
| #01 – Does the written informed consent form include information about the diagnosis or underlying condition? | | ☐ ☐ ☐ ☐ ☐ *1 2 3 4 5 No Partially Yes* | | |
| #02 – Does it describe the nature of the recommended intervention? | | ☐ ☐ ☐ ☐ ☐ *1 2 3 4 5 No Partially Yes* | | |
| #03 – Does it describe the purpose (or goal) of the recommended intervention? | | ☐ ☐ ☐ ☐ ☐ *1 2 3 4 5 No Partially Yes* | | |
| #04 – Are the possible risks of the recommended intervention sufficiently addressed? | | ☐ ☐ ☐ ☐ ☐ *1 2 3 4 5 No Partially Yes* | | |
| #05 – Is it made clear that alternative interventions (including the option of forgoing any kind of intervention) are available? | | ☐ ☐ ☐ ☐ ☐ *1 2 3 4 5 No Partially Yes* | | |
| #06 – Does the written informed consent form include information about the reasons why the recommended intervention is preferred over the available alternatives? | | ☐ ☐ ☐ ☐ ☐ *1 2 3 4 5 No Partially Yes* | | |
| #07 – Does it include basic information about the risks as well as possible benefits of all other options? | | ☐ ☐ ☐ ☐ ☐ *1 2 3 4 5 No Partially Yes* | | |
| #08 – Does it describe the possible consequences of forgoing any kind of intervention? | | ☐ ☐ ☐ ☐ ☐ *1 2 3 4 5 No Partially Yes* | | |
| #09 – Are instructions regarding the appropriate peri-interventional behavior included? | | ☐ ☐ ☐ ☐ ☐ *1 2 3 4 5 No Partially Yes* | | |
| #10 – Does it inform the reader about the option of asking the health care provider for additional information? | | ☐ ☐ ☐ ☐ ☐ *1 2 3 4 5 No Partially Yes* | | |
| #11 – Based on the ratings to all of the above items, how would you rate the overall impression of this written informed consent form? | | ☐ ☐ ☐ ☐ ☐ *1 2 3 4 5 Negative Moderate Positive* | | |
| **Total score (i.e., the sum of items #1 to #11)** | | **Interpretation** | | |
| 11-19 points  20-28 points  29-37 points  38-46 points  47-55 points | | | Very poor  Poor  Fair  Good  Excellent | |

**Supplemental file 2.** Detailed description of the Graz Assessment Tool of Written Informed Consent Keypoints (GATWICK).

| **Quality of Informed Consent Forms (ICFs) measured through GATWICK score, median (interquartile range)** | | | | | |  |
| --- | --- | --- | --- | --- | --- | --- |
| ***Overall quality*** | |  | |  | | |
| Conventional (i.e., web-based) ICFs  AI-generated ICFs  *All combined* | | 27.50 (16.9)  32.50 (8.3)  *31.00 (14.0)* | | *p* = 0.007* | | |
| ***Differences by procedure*** |  | | |  | | |
| Apicoectomy  Biopsy  Oral bone augmentation  Dental cystectomy  Dental implants  Oral incision and drainage  Dental local anesthesia  Periodontal surgery  Tooth extraction  Wisdom tooth removal | | 27.00 (13.1)  30.50 (7.3)  31.50 (11.8)  31.25 (10.9)  33.25 (16.5)  31.50 (16.0)  28.50 (13.5)  36.50 (7.3)  23.50 (12.8)  28.25 (16.9) | | *p* = 0.004^†^ | | |
| ***Differences by Large Language Model*** | | |  |  | | |
| *ChatGPT Claude*  *Bing Chat*  *Google Bard* | | 34.25 (4.0)  40.50 (8.0)  30.00 (4.5)  26.50 (14.0) | | | *p* < 0.001^†^ | |

**Supplemental file 3.** AI-generated informed consent forms performed significantly better than conventional versions, with notable differences across oral surgical procedures and among the types of large language models used (^*^ = Mann-Whitney U-test; ^†^ = Kruskal-Wallis test).
